# Supplementary material for: Home interventions and light therapy for the treatment of vitiligo (HI-Light Vitiligo Trial): study protocol for a randomised controlled trial
Source: BMJ Open. 2018 Apr 3;8(4):e018649. doi: 10.1136/bmjopen-2017-018649 (PMC5893933; doi:10.1136/bmjopen-2017-018649)
Supplement: Supplementary file 1 [file bmjopen-2017-018649supp001.pdf]

Appendix 1. Treatment Schedule

Step	Nominal Dose (J/cm²)	Treatment Duration	Step	Nominal Dose (J/cm²)	Treatment Duration
1	0.05	00:15	20	0.56	02:43
2	0.1	00:29	21	0.61	02:59
3	0.11	00:32	22	0.67	03:17
4	0.12	00:35	23	0.74	03:36
5	0.13	00:39	24	0.81	03:58
6	0.15	00:43	25	0.90	04:22
7	0.16	00:47	26	0.98	04:48
8	0.18	00:52	27	1.08	05:17
9	0.19	00:57	28	1.19	05:48
10	0.21	01:03	29	1.31	06:23
11	0.24	01:09	30	1.44	07:02
12	0.26	01:16	31	1.59	07:44
13	0.29	01:23	32	1.74	08:30
14	0.31	01:32	33	1.92	09:21
15	0.35	01:41	34	2.11	10:17
16	0.38	01:51	35	2.32	11:19
17	0.42	02:02	36	2.55	12:27
18	0.46	02:14	37	2.81	13:42
19	0.51	02:28			
