# Supplementary material for: Home interventions and light therapy for the treatment of vitiligo (HI-Light Vitiligo Trial): study protocol for a randomised controlled trial
Source: BMJ Open. 2018 Apr 3;8(4):e018649. doi: 10.1136/bmjopen-2017-018649 (PMC5893933; doi:10.1136/bmjopen-2017-018649)
Supplement: Supplementary file 2 [file bmjopen-2017-018649supp002.pdf]

Appendix 2. Summary of Instructions for adjusting light therapy treatment schedule and dosing

Situation	What to Do
No erythema or side effects after last treatment	Increase your dose by one step for the next treatment.
**Erythema or Overdose**	
Grade 1 erythema after last treatment	Go back one step on treatment schedule for next treatment
Grade 2 erythema after last treatment	Skip next scheduled treatment. Go back one step on treatment schedule for following treatment.
Grade 3 erythema or 4 erythema after last treatment	Apply thick layer of trial ointment and contact local research team or local on-call dermatologist. Treatment to resume only on advice of local research team.
Light Overdose (used for 20% longer or more than intended treatment time)	Apply thick layer of trial ointment and seek medical attention (prescription for clobetasol propionate 0.05% twice a day for 2-3 days required). Treatment to resume only on advice of local research team.
**Missed Treatments**	
One or two missed treatments	At next session, go back one step on treatment schedule.
Three missed treatments	At next session, go back two steps on treatment schedule.
Four or more missed treatments	Contact local research team for advice on new starting dose[1].
**Side Effects**	
Itchy or dry skin	Apply moisturiser 3-4 times a day, but not within 2 hours before light treatment. Continue treatments as normal.
Tan around edges	This is normal. Continue treatments as normal.
Rash	Stop treatment immediately and seek medical advice. Treatment to resume only on advice of local research team.
Cold sore	Stop light treatment until the cold sore has healed. Adjust next treatment time according to missed treatment advice.

[1] Sites give following advice: for 4-6 missed treatments, reduce the next dose to 50% of the last given dose. For more than 6 missed treatments, the treatment schedule should be restarted from the beginning.
